# Supplementary material for: New criteria for sepsis-induced coagulopathy (SIC) following the revised sepsis definition: a retrospective analysis of a nationwide survey
Source: BMJ Open. 2017 Sep 27;7(9):e017046. doi: 10.1136/bmjopen-2017-017046 (PMC5623518; doi:10.1136/bmjopen-2017-017046)
Supplement: Author's manuscript [file bmjopen-2017-017046.draft_revisions.pdf]

BMJ Open

BMJ Open is committed to open peer review. As part of this commitment we make the peer review history of every article we publish publicly available.

When an article is published we post the peer reviewers' comments and the authors' responses online. We also post the versions of the paper that were used during peer review. These are the versions that the peer review comments apply to.

The versions of the paper that follow are the versions that were submitted during the peer review process. They are not the versions of record or the final published versions. They should not be cited or distributed as the published version of this manuscript.

BMJ Open is an open access journal and the full, final, typeset and author-corrected version of record of the manuscript is available on our site with no access controls, subscription charges or pay-per-view fees (http://bmjopen.bmj.com).

If you have any questions on BMJ Open's open peer review process please email info.bmjopen@bmj.com

BMJ Open

New Criteria for Sepsis-induced coagulopathy (SIC) following the revised sepsis definition

Journal:	*BMJ Open*
Manuscript ID	bmjopen-2017-017046
Article Type:	Research
Date Submitted by the Author:	15-May-2017
Complete List of Authors:	Iba, Toshiaki; Juntendo University Graduate School of Medicine, Department of Emergency and Disaster Med<br>Di Nisio, Marcello; University "G. D'Annunzio" of Chieti-Pescara, Department of Medical, Oral and Biotechnological Sciences; Academic Medical Center, Department of Vascular Medicine<br>Levy, Jerrold; Duke University, Anesthesiology<br>Kitamura, Naoya; Asahi Kasei Pharma Corporation, Pharmaceuticals Sales Division<br>Thachil, Jecko; Manchester Royal Infirmary
**Primary Subject Heading**:	Emergency medicine
Secondary Subject Heading:	Diagnostics
Keywords:	disseminated intravascular coagulation, prothrombin time, platelet count, sepsis, thrombomodulin

SCHOLARONE™
Manuscripts

For peer review only

2017/3/27

Research article

New Criteria for Sepsis-induced coagulopathy (SIC) following the revised sepsis definition

Toshiaki Iba, M.D.[1]; Marcello Di Nisio, M.D.[2]; Jerrold H. Levy, M.D.[3]; Naoya Kitamura, B.S.[4]; and Jecko Thachil, M.D.[5]

[1] Professor of Emergency and Disaster Medicine, Juntendo University Graduate School of Medicine, Tokyo, Japan, toshiiba@cf6.so-net.ne.jp

[2] Professor of Medical, Oral and Biotechnological Sciences, University G D'Annunzio of Chieti-Pescara, Chieti, Italy, mdinisio@unich.it

[3] Professor of Anesthesiology and Surgery, Duke University School of Medicine, Durham, NC, jerrold.levy@duke.edu

[4] Chief of Recomodulin Strategy Planning Department, Pharmaceuticals Sales Division, Asahi Kasei Pharma Corporation, Tokyo, Japan, kitamura.nb@om.asahi-kasei.co.jp

[5] Professor of Haematology, Manchester Royal Infirmary, Manchester, UK, Jecko.thachil@cmft.nhs.uk

For peer review only - http://bmjopen.bmj.com/site/about/guidelines.xhtml

Corresponding author: Toshiaki Iba, MD.

Professor of Emergency and Disaster Medicine, Juntendo University Graduate School of Medicine

2-1-1 Hongo Bunkyo-ku, Tokyo 113-8421, Japan

E-mail: toshiiba@cf6.so-net.ne.jp, Tel: 81-3-3813-3111 (X: 5818) Fax: 81-3-3813-5431

Strength of this study

- Sepsis-induced coagulopathy (SIC) was the first criterion specifically designed for sepsis-associated DIC following the revised sepsis definition (Sepsis-3).
- SIC is newly designed to select a possible candidate for the anticoagulant therapy among the patients with sepsis and coagulopathy.
- SIC is defined by the platelet count, PT ratio and SOFA score. It is easy to use and provides important information.
-

Limitations

- All the subjects utilized in this study were treated with recombinant thrombomodulin. Therefore, SIC might applicable for the patients who are going to be treated with this agent. Further evaluation of the prognostic value of the SIC sore in patients not receiving any anticoagulant treatment is required.

Short title: Proposal of new criteria for sepsis and coagulopathy

For peer review only

Abstract

OBJECTIVE: Recent clinical studies have shown that anticoagulant therapy might be effective only in specific at-risk subgroups of patients with sepsis and coagulation dysfunction. The definition of sepsis was recently modified and old scoring systems may no longer be proper for the diagnosis of sepsis-associated coagulopathy. The aim of this study was to evaluate prognostic factors in patients diagnosed as sepsis and coagulopathy according to the new sepsis definition and assess their accuracy in comparison to existing models.

DESIGN, SETTING AND PARTICIPANTS: We evaluated the prognostic value of the newly proposed diagnostic criteria for sepsis-induced coagulopathy. The data set was obtained from a post-marketing survey examining recombinant human soluble thrombomodulin and offered by Japanese Society on Thrombosis and Hemostasis. A total of 1498 Japanese patients with sepsis and coagulopathy complications who were treated with recombinant thrombomodulin were analyzed in this study.

MAIN OUTCOME MEASURES: The platelet count, prothrombin time (PT) ratio, fibrinogen and fibrin degradation products (FDP), systemic inflammatory response syndrome (SIRS) score, and sequential organ failure assessment (SOFA) score obtained just before the start of treatment were examined in relation to the 28-day mortality rate.

RESULTS: The platelet count, PT ratio, and total SOFA were independent predictors of a fatal outcome in a logistic regression model. A sepsis-induced coagulation (SIC) score was

For peer review only - http://bmjopen.bmj.com/site/about/guidelines.xhtml

defined using the 3 above-mentioned variables with a positivity threshold of 4 points or more. The SIC score predicted higher 28-day mortality rate compared to the Japanese Association for Acute Medicine (JAAM)-DIC score (38.4% vs. 34.7%).

CONCLUSIONS: The SIC scoring is based on readily available parameters, is easy to calculate and owns a high predictive value for 28-day mortality. Future studies are warranted to evaluate whether the SIC score may guide the decision to initiate anticoagulant therapy.

Keywords

disseminated intravascular coagulation; prothrombin time; platelet count; sepsis; thrombomodulin

For peer review only - http://bmjopen.bmj.com/site/about/guidelines.xhtml

Introduction

Shock and disseminated intravascular coagulation (DIC) are the two major causes of organ dysfunction in sepsis [1]. Dhainaut et al. reported that DIC is a strong predictor of mortality independent of the severity of sepsis [2]. As for the management of septic shock, advances have been made in recent years leading to significant improvements in survival [3]. In contrast, much less attention has been paid to DIC [4].

Although diagnostic criteria exist for DIC, none were specially designed for sepsis-associated DIC [5-7] that is uniquely characterized by coagulation activation with over-suppression of fibrinolysis and a high incidence of organ dysfunction [8]. The objectives of diagnostic criteria should identify a homogenous group of patients with the same basic pathophysiology and clinical characteristics, and improve patient outcomes by providing specific treatment interventions [9]. To reach these goals, diagnostic criteria should meet the following three conditions: (1) they should be readily available and easy to use; (2) they should enable diagnostic accuracy; and (3) they should have prognostic value. As a new definition for sepsis was announced in 2016 [10], a DIC score that matches the new sepsis definition and may identify patients who would benefit from anticoagulant treatment is urgently needed. Therefore, the aim of this study is to identify the patients who were estimated to achieve the benefit from the anticoagulant therapy.

Methods

For peer review only - http://bmjopen.bmj.com/site/about/guidelines.xhtml

Data collection

The data set was obtained from a post-marketing survey examining recombinant human soluble thrombomodulin (TM-α; Asahi Kasei Parma Corporation, Tokyo, Japan) performed by Asahi Kasei Pharma Corporation between May 2008 and March 2010 [11] and kindly provided by the Japanese Society on Thrombosis and Hemostasis with permission. All of the cases treated in Japan during this period were registered in the survey. The survey was conducted in accordance with the Declaration of Helsinki and Good Vigilance Practice and Good Post-marketing Study Practice. Since the anonymization of personal data was performed upon data collection, the ethical committees waived the need for informed consent acquisition.

At the time of data collection, sepsis was defined according to the American College of Chest Physicians/Society of Critical Care Medicine consensus definition [12]. A total of 2516 Japanese patients with sepsis-associated coagulation disorder were registered in this survey; however, since the record of sequential organ failure assessment (SOFA) data was not mandatory, a complete data set was only obtained in 1498 cases, and all of these patients were analyzed in this study. All patients were treated with TM-α (0.06 mg/kg/day for 6 days) by either intravenous bolus injection or intravenous infusion (diluted in 50 mL 0.9% saline) over 15 minutes via a catheter; the exclusion criteria were as follows: age of less than 18 years, major bleeding, systemic inflammatory response syndrome (SIRS) score ≦ 1, hypersensitivity to TM-α, and pregnancy.

For peer review only - http://bmjopen.bmj.com/site/about/guidelines.xhtml

Laboratory measurements and organ dysfunction assessments

Blood samples obtained just before the initiation of anticoagulant therapy were analyzed and the data were defined as initial data. The platelet count, fibrinogen/fibrin degradation products (FDP), and prothrombin time (PT)-INR (international normalization ratio) were measured in local laboratories. The Japanese Association for Acute Medicine (JAAM)-DIC score [7] was calculated based on the initial (just before the treatment) laboratory data and the SIRS score. Respiratory dysfunction, cardiovascular dysfunction, and hepatic and renal dysfunction were assessed using the SOFA scores [13], and a score of 2 or more was defined as organ dysfunction.

Statistical analysis

Differences in patient characteristics between survivors and non-survivors were examined using the Fisher exact test or unpaired Wilcoxon signed-rank test. Then, the relationships between the 28-day mortality rate and the initial data were examined in univariate and multivariate analyses using logistic regression analysis (the enter method). The analysis was conducted using the outcome (survived, 0; died, 1) as the dependent variable and age, sex, SIRS score, platelet count, PT ratio, and the proportion of various organ dysfunction as explanatory variables.

The numerical values in the text and tables represent the median and interquartile range (IQR), unless otherwise noted. The results of the logistic regression analysis were reported as the odds ratio (OR), 95% confidence interval (CI) and *P*-values. For all the reported

For peer review only - http://bmjopen.bmj.com/site/about/guidelines.xhtml

results, a value of $P < 0.05$ was considered to denote statistical significance. The above-mentioned analyses were performed using JMP software, version 9.0 (SAS Institute Co., Ltd., Cary, North Carolina).

Results

Baseline characteristics

Among the 1498 patients, 994 (66.4%) survived at 28 days and 504 (33.6%) died. Table 1 shows the baseline characteristics of the patients. The median age was lower and the proportion of women was larger among the survivors. No difference in the SIRS score (P = 0.0556) was seen between the two groups, but the JAAM-DIC score (P = 0.0019) was higher among the non-survivors. Regarding the hemostatic parameters, the platelet count was higher ($P < 0.0001$) and the PT ratio was lower ($P < 0.0001$) among the survivors. In contrast, the FDP level was not significantly different between the survivors and non-survivors. The initial total SOFA score was significantly higher among the non-survivors ($P < 0.0001$), and the proportions of patients with respiratory dysfunction ($P < 0.0001$), cardiovascular dysfunction (P = 0.0426), hepatic dysfunction ($P < 0.0001$), or renal dysfunction ($P < 0.0001$) were higher among the non-survivors.

Relationships between biomarkers and mortality

The results of the univariate and multivariable analyses are shown in Table 2. A multivariate analysis using the enter method showed that patient age (P = 0.002), a male sex (P = 0.017), a decreased platelet count (P = 0.005), the higher PT ratio (P = 0.024), and

For peer review only - http://bmjopen.bmj.com/site/about/guidelines.xhtml

higher total SOFA ($P < 0.001$) were significantly and independently associated with the 28-day mortality rate. In contrast, the initial SIRS score was not correlated with survival (Table 2).

The SIC score

The SIC score was developed based on the result of the logistic regression analyses. The relationship between the initial platelet count and mortality is shown in Figure 1, left panel. As shown in the figure, the mortality rate was less than 30% when the platelet count was more than 100 x 10^3 μL but increased to 35% when the platelet count decreased to below 100 x 10^3 μL. In contrast, the mortality rate increased steadily as the initial PT ratio increased and reached 40% at a PT ratio of more than 1.4 (Figure 1, middle). The mortality increased along with the increase of initial total SOFA (Figure 1, right). For the SIC scoring, the organ dysfunction scores were defined according to the criteria used for SOFA scoring [13]. Total SOFA was scored based on the scores of respiratory SOFA, cardiovascular SOFA, hepatic SOFA and renal SOFA. The score of total SOFA was defined as 2 if the total score exceeded 2. For the PT ratio, the cutoff value to assign 1 point was set at 1.2 in accordance with the JAAM-DIC criteria, while the cutoff value for 2 points was set at 1.4 based on the case number distribution and the mortality rate. Finally, SIC was defined as a total score of 4 or more in Table 3, since the mortality rate at this point exceeded 20% (Figure 2, left), with the requirement that the total score for the platelet count and the PT ratio exceed 2.

For peer review only - http://bmjopen.bmj.com/site/about/guidelines.xhtml

Comparison of the SIC and JAAM-DIC criteria

A total of 902 patients were diagnosed as having SIC, while 1332 patients met the JAAM criteria for DIC. The respective mortality rates for these classifications were 38.4% and 34.7% (Table 4). Figure 2 shows the relationship between the 28-day mortality rate and the SIC scores (left panel) and the JAAM-DIC score (right panel). Using the SIC scoring system, the mortality rate increased in a linear fashion. The mortality rate was 30% at a score of 4 and increased steeply to a maximum of over 45% at a score of 6. In contrast, the mortality rate did not seem to be strongly correlated with the JAAM-DIC score. The mortality rate had already exceeded 20% at scores of 1 to 3, and it increased to over 30% at a score of 4. The mortality rate gradually increased thereafter, reaching approximately 40% at a score of 8.

Discussion

The relationship and balance between coagulation and fibrinolysis system is regulated by a complex series of interactions. Microbial products induce the synthesis and the release of inflammatory mediators during sepsis. In addition to these pathogen-associated molecular patterns (PAMPs), pro-inflammatory substances known as damage-associated molecular patterns (DAMPs) are also released from activated or damaged host cells [14,15]. Both PAMPs and DAMPs activate the coagulation system together with the host immune response, leading to DIC [14,16]. Since the primary function of these biologic responses is

For peer review only - http://bmjopen.bmj.com/site/about/guidelines.xhtml

to assist the host in sequestering and eliminating the microbes, the suppression of coagulation at this stage may not lead to a better outcome. In contrast, patients with compromised organ circulation may benefit of anticoagulant therapy [17]. However, all of the anticoagulants examined in the early 21st century failed to show any efficacy [18,19]. One of the major reasons for this was that these studies targeted severe sepsis, rather than sepsis-associated coagulopathy [2,20]. After a series of disappointing reports, Umemura et al. suggested that anticoagulant therapies might be beneficial if they were applied to septic patients with a severe coagulation disorder [21]. In addition, Yamakawa et al. also reported an association between the application of anticoagulant therapy and a decrease in mortality among patients with a high risk of death (SOFA score of 13–17) [22]. With respect to recombinant thrombomodulin, recent studies examining its effects in patients with septic DIC have repeatedly reported favorable results [2,22] with more prominent benefits as the severity increased [23,24]. In a Phase 2 clinical trial performed in 17 countries, recombinant thrombomodulin tended to exhibit an effect in patients with organ dysfunction having a PT ratio of greater than 1.4 before the treatment [24]. Recombinant thrombomodulin has been used in patients who meet the JAAM-DIC criteria in Japan, which, however, might not be appropriate [25]. Yoshimura et al [26]. reported that a beneficial effect was only seen in patients diagnosed as having DIC and who had an APACHE II score of between 24 and 29. Thus, to identify appropriate candidates for anticoagulant therapy, we added a category for organ dysfunction in patients with SIC and

For peer review only - http://bmjopen.bmj.com/site/about/guidelines.xhtml

targeted individuals with a mortality rate higher than that of patients meeting the JAAM-DIC criteria. This new SIC category represents "sepsis (Third International Consensus Definitions [Sepsis 3] [10]), and coagulation disorder," which could be a suitable target for therapeutic interventions. Previous reports have consistently reported efficacy when the 30-day mortality rate in the treated group was between 20% and 30% [27,28], and not below 20% [24]. As a matter of fact, a subgroup analysis of a Phase 3 study performed in Japan demonstrated that the mortality rate in a heparin-control group was 31.6%, while that of a TM-α group was 21.4% [29]. In the present study, the mortality rate of the SIC group was about 30% when the total score was 4, and it increased as the SIC score increased.

Another purpose of this study was to compare the SIC and JAAM-DIC classifications. In SIC, the SIRS score used in the JAAM-DIC criteria was replaced with the SOFA score, and the FDP criterion was eliminated. The prognostic relevance of SIRS has been questioned and it was not used in the new definition of sepsis. The significance of fibrin-related markers in DIC differs depending on the underlying disease [30], and the impact of the FDP criterion was limited in patients with fibrinolysis-suppressed-type coagulation disorders, as represented by sepsis. The current study indicated that the SIC score identified patients with a higher risk of death, compared with the JAAM-DIC score (38.4% vs. 34.7%), and the numbers of diagnosed cases were 902 and 1332, respectively. Therefore, we speculate that the SIC score might better identify candidates for

For peer review only - http://bmjopen.bmj.com/site/about/guidelines.xhtml

anticoagulant therapy rather than the JAAM-DIC score. The other advantage of SIC is the simplicity of its calculation. Indeed, the FDP criterion was omitted, and SIRS score was replaced to SOFA score. Since the SOFA score is routinely evaluated in the ICU, the addition of a PT test is relatively easy to implement in clinical practice.

Limitations

There are limitations to our current study. First, data from a post-marketing survey was utilized in this study. Thus, all the subjects were treated with recombinant thrombomodulin. Data from other databases of patients treated without anticoagulants, or treated with other anticoagulants, such as antithrombin, should also be examined. Second, the goal of this study was to identify a subgroup of patients with high mortality rates who could benefit of anticoagulant therapy. Further evaluation of the prognostic value of the SIC sore in patients not receiving any anticoagulant treatment is warranted.

Conclusions

We have proposed SIC as a new category of patients with "sepsis and coagulopathy." Since the SIC category adheres to the new sepsis criteria (Third International Consensus Definitions), this definition will be easy to use and should provide important and novel information to the physicians.

For peer review only

For peer review only - http://bmjopen.bmj.com/site/about/guidelines.xhtml

Acknowledgement

The authors would like to thank all the institutes that participated in the post-marketing surveillance.

Contributors

TI, MDN, and JT conceived of the study, and participated in its design. TI and MDN participated in the sequence alignment and drafted the manuscript. JL helped to revise the manuscript. NK helped to collect and arrange the data. All authors read and approved the final manuscript.

Funding

This work was supported by Ministry of Education, Culture, Sports, Science and Technology-Supported Program for the Strategic Research Foundation at Private Universities 2016.

Competing interests

KN is an employee of Asahi Kasei Pharma Corporation. The other authors have no competing interests to declare.

Data sharing statement

For peer review only

For peer review only - http://bmjopen.bmj.com/site/about/guidelines.xhtml

Full data and data analysis files are available on request.

Supplementary resources

None

For peer review only

For peer review only - http://bmjopen.bmj.com/site/about/guidelines.xhtml

References

1. Gando S, Levi M, Toh CH: **Disseminated intravascular coagulation.** *Nat Rev Dis Primers* 2016, **2**:16037.

2. Dhainaut JF, Yan SB, Joyce DE, Pettilä V, Basson B, Brandt JT, Sundin DP, Levi M: **Treatment effects of drotrecogin alfa (activated) in patients with severe sepsis with or without overt disseminated intravascular coagulation.** *J Thromb Haemost* 2004, **2**:1924-1933.

3. Kadri SS, Rhee C, Strich JR, Morales MK, Hohmann S, Menchaca J, Suffredini AF, Danner RL, Klompas M: **Estimating Ten-Year Trends in Septic Shock Incidence and Mortality in United States Academic Medical Centers Using Clinical Data.** *Chest* 2016, **151**(2):278-285.

4. Dellinger RP, Levy MM, Rhodes A, Annane D, Gerlach H, Opal SM, Sevransky JE, Sprung CL, Douglas IS, Jaeschke R, *et al*: **Surviving sepsis campaign: international guidelines for management of severe sepsis and septic shock: 2012.** *Crit Care Med* 2013; **41**:580-637.

5. Kobayashi N, Maekawa T, Takada M, Tanaka H, Gonmori H: **Criteria for diagnosis of DIC based on the analysis of clinical and laboratory findings in 345 DIC patients collected by the Research Committee on DIC in Japan.** *Bibl Haematol* 1983, **49**:265-275.

For peer review only - http://bmjopen.bmj.com/site/about/guidelines.xhtml

6. Taylor FB Jr, Toh CH, Hoots WK, Wada H, Levi M: **Towards definition, clinical and laboratory criteria, and a scoring system for disseminated intravascular coagulation.** *Thromb Haemost* 2001, **86**:1327-1330.

7. Gando S, Iba T, Eguchi Y, Ohtomo Y, Okamoto K, Koseki K, Mayumi T, Murata A, Ikeda T, Ishikura H, et al: **A multicenter, prospective validation of disseminated intravascular coagulation diagnostic criteria for critically ill patients: comparing current criteria.** *Crit Care Med* 2006, **34**:625-631.

8. Levi M, Ten Cate H: **Disseminated intravascular coagulation.** *N Engl J Med* 1999, **341**:586-592.

9. Gando S, Meziani F, Levi M: **What's new in the diagnostic criteria of disseminated intravascular coagulation?** *Intensive Care Med* 2016; **42**:1062-1064.

10. Singer M, Deutschman CS, Seymour CW, Shankar-Hari M, Annane D, Bauer M, Bellomo R, Bernard GR, Chiche JD, Coopersmith CM, *et al*: **The Third International Consensus Definitions for Sepsis and Septic Shock (Sepsis-3).** *JAMA* 2016, **315**:801-810.

11. Mimuro J, Takahashi H, Kitajima I, Tsuji H, Eguchi Y, Matsushita T, Kuroda T, Sakata Y: **Impact of recombinant soluble thrombomodulin (thrombomodulin alfa) on disseminated intravascular coagulation.** *Thromb Res* 2013, **131**:436-443.

12. Bone RC, Balk RA, Cerra FB, Dellinger RP, Fein AM, Knaus WA, Schein RM, Sibbald WJ: **Definitions for sepsis and organ failure and guidelines for the use of**

For peer review only - http://bmjopen.bmj.com/site/about/guidelines.xhtml

innovative therapies in sepsis. The ACCP/SCCM Consensus Conference Committee. American College of Chest Physicians/Society of Critical Care Medicine. *Chest* 1992, **101**:1644-1655.

13. Vincent JL, de Mendon.a A, Cantraine F, Moreno R, Takala J, Suter PM, Sprung CL, Colardyn F, Blecher S: **Use of the SOFA score to assess the incidence of organ dysfunction/failure in intensive care units: results of a multicenter, prospective study.** *Crit Care Med* 1998, **26**:1793-1800.
14. Esmon CT, Xu J, Lupu F: **Innate immunity and coagulation.** *J Thromb Haemost* 2011, **9**:182-188.
15. Seong SY, Matzinger P: **Hydrophobicity: an ancient damage-associated molecular pattern that initiates innate immune responses.** *Nat Rev Immunol* 2004, **4**: 469-**4**78.
16. Liaw PC, Ito T, Iba T, Thachil J, Zeerleder S: **DAMP and DIC: The role of extracellular DNA and DNA-binding proteins in the pathogenesis of DIC.** *Blood Rev* 2016, **30**:257-261.
17. Schouten M, Wiersinga WJ, Levi M, van der Poll T: **Inflammation, endothelium, and coagulation in sepsis.** *J Leukoc Biol* 2008, **83**:536-545.
18. Warren BL, Eid A, Singer P, Pillay SS, Carl P, Novak I, Chalupa P, Atherstone A, Pénzes I, Kübler A, et al: **Caring for the critically ill patient. High-dose antithrombin III in severe sepsis: a randomized controlled trial.** *JAMA* 2001, **286**:1869-1878.

For peer review only - http://bmjopen.bmj.com/site/about/guidelines.xhtml

19. Ranieri VM, Thompson BT, Barie PS, Dhainaut JF, Douglas IS, Finfer S, Gårdlund B, Marshall JC, Rhodes A, Artigas A, et al: **Drotrecogin alfa (activated) in adults with septic shock.** *N Engl J Med* 2012, **366**:2055-2064.

20. Kienast J, Juers M, Wiedermann CJ, Hoffmann JN, Ostermann H, Strauss R, Keinecke HO, Warren BL, Opal SM: **Treatment effects of high-dose antithrombin without concomitant heparin in patients with severe sepsis with or without disseminated intravascular coagulation.** *J Thromb Haemost* 2006, **4**:90-97.

21. Umemura Y, Yamakawa K, Ogura H, Yuhara H, Fujimi S: **Efficacy and safety of anticoagulant therapy in three specific populations with sepsis: a meta-analysis of randomized controlled trials.** *J Thromb Haemost* 2015, **14**:518-530.

22. Yamakawa K, Umemura Y, Hayakawa M, Kudo D, Sanui M, Takahashi H, Yoshikawa Y, Hamasaki T, Fujimi S: **Benefit profile of anticoagulant therapy in sepsis: a nationwide multicentre registry in Japan.** *Crit Care* 2016, **20**:229.

23. Yamakawa K, Aihara M, Ogura H, Yuhara H, Hamasaki T, Shimazu T: **Recombinant human soluble thrombomodulin in severe sepsis: a systematic review and meta-analysis.** *J Thromb Haemost* 2015, **13**:508-519.

24. Vincent JL, Ramesh MK, Ernest D, LaRosa SP, Pachl J, Aikawa N: **A randomized, double-blind, placebo-controlled, Phase 2b study to evaluate the safety and efficacy of recombinant human soluble thrombomodulin, ART-123, in patients with sepsis and suspected disseminated intravascular coagulation.** *Crit Care Med* 2013, **41**:2069-2079.

For peer review only - http://bmjopen.bmj.com/site/about/guidelines.xhtml

25. Tagami T, Matsui H, Horiguchi H, Fushimi K, Yasunaga H: **Recombinant human soluble thrombomodulin and mortality in severe pneumonia patients with sepsis-associated disseminated intravascular coagulation: an observational nationwide study.** *J Thromb Haemost* 2015, **13**:31-40.

26. Yoshimura J, Yamakawa K, Ogura H, Umemura Y, Takahashi H, Morikawa M, Inoue Y, Fujimi S, Tanaka H, Hamasaki T, et al: **Benefit profile of recombinant human soluble thrombomodulin in sepsis-induced disseminated intravascular coagulation: a multicenter propensity score analysis.** *Crit Care* 2015, **19**:78.

27. Yamakawa K, Ogura H, Fujimi S, Morikawa M, Ogawa Y, Mohri T, Nakamori Y, Inoue Y, Kuwagata Y, Tanaka H, et al: **Recombinant human soluble thrombomodulin in sepsis-induced disseminated intravascular coagulation: a multicenter propensity score analysis.** *Intensive Care Med* 2013, **39**:644-652.

28. Hayakawa M, Yamakawa K, Saito S, Uchino S, Kudo D, Iizuka Y, Sanui M, Takimoto K, Mayumi T, Ono K: **Recombinant human soluble thrombomodulin and mortality in sepsis-induced disseminated intravascular coagulation. A multicentre retrospective study.** *Thromb Haemost* 2016, **115**:1157-66.

29. Aikawa N, Shimazaki S, Yamamoto Y, Saito H, Maruyama I, Ohno R, Hirayama A, Aoki Y, Aoki N: **Thrombomodulin alfa in the treatment of infectious patients complicated by disseminated intravascular coagulation: subanalysis from the phase 3 trial.** *Shock* 2011, **35**:349-354.

For peer review only - http://bmjopen.bmj.com/site/about/guidelines.xhtml

30. Wada H, Matsumoto T, Yamashita Y, Hatada T: **Disseminated intravascular coagulation: testing and diagnosis.** *Clin Chim Acta* 2014, **436**:130-134.

For peer review only

For peer review only - http://bmjopen.bmj.com/site/about/guidelines.xhtml

Legends

Figure 1. Patient counts and mortality rates according to platelet count, prothrombin time ratio, and total SOFA score. The bar graph shows the number of patients in each category, and the line graph represents the mortality rate. X-axis represents the score and (case number). Left: Many of the patients had an initial platelet count of 100 x 10^3 µL or less. Mortality increased to 35% when the count decreased to less than 100 x 10^3 µL. Middle: Mortality increased along with an increase in the prothrombin time ratio, reaching more than 40% when the prothrombin time ratio was more than 1.4. Right: The population of total SOFA score of 0 and 1 are quite limited, and the mortality of this population was lower than that of the score of 2 or more.

Figure 2. Patient counts and mortality rates according to the sepsis-induced coagulopathy (SIC) and the Japanese Association for Acute Medicine (JAAM) disseminated intravascular coagulation (DIC) classifications. The patient distributions (bars) and the mortality rates (lines) are plotted according to the SIC scores (left) and the JAAM-DIC scores (right). X-axis represents the score and (case number). The mortality rate increased as SIC score elevated and exceeded 20% at a score of 4. In contrast, the mortality rate exceeded 30% at a JAAM-DIC score of 4 and gradually increased to 40%.

For peer review only - http://bmjopen.bmj.com/site/about/guidelines.xhtml

Table 1. Baseline characteristics of the patients

Characteristics	Survivor (n = 994)	Non-survivor (n = 504)	*P*-value
Age (years)	70 (58–78)	73 (62–80)	0.0001
Sex (male/female)	560/434	329/175	0.0009
Baseline values			
SIRS score	3 (2–4)	3 (2–4)	0.0556
SIRS score ≧3	625(62.9%)	341(67.7%)	0.0677
JAAM-DIC score	5 (4–7)	6 (5–7)	0.0019
Platelet count (x 10^3 μL)	61 (36–89)	49 (29–78)	< 0.0001
FDP (μg/mL)	25.3 (13.0–51.9)	25.4 (12.2–51.7)	0.7788
PT ratio	1.30 (1.16-1.48)	1.36 (1.21–1.64)	< 0.0001
Organ dysfunction			
Total SOFA	5(3–6)	5 (4–7)	< 0.0001
respiratory SOFA ≧2	621 (62.5%)	395 (78.4%)	< 0.0001
cardiovascular SOFA ≧2	636 (64.0%)	349 (69.3%)	0.0426
hepatic SOFA ≧2	272 (27.4%)	202 (40.8%)	< 0.0001
renal SOFA ≧2	326 (32.8%)	221 (43.8%)	< 0.0001

JAAM Japanese association for acute medicine, *DIC* disseminated intravascular coagulation, *SIRS* systemic inflammatory response syndrome, *FDP* fibrinogen and fibrin degradation products, *PT* prothrombin time, *SOFA* sequential organ failure assessment

Total SOFA is scored by 4 items (respiratory SOFA, cardiovascular SOFA, hepatic SOFA, renal SOFA). The score of each organ is defined as 2 in case of 2 or more.

For peer review only - http://bmjopen.bmj.com/site/about/guidelines.xhtml

Table 2. Relationship between 28-day mortality and baseline characteristics

Item	Univariate			Multivariate		
	OR	95% CI	*P*-value	OR	95% CI	*P*-value
Age (years)	1.010	1.004-1.016	0.002	1.010	1.004-1.017	0.002
Sex (male/female)	1.457	1.168-1.821	0.001	1.323	1.052-1.668	0.017
Platelet count (x10^9/L)	0.965	0.945-0.984	0.000	0.972	0.951-0.992	0.005
PT ratio	1.225	1.065-1.435	0.004	1.169	1.020-1.364	0.024
Total SOFA	1.252	1.181-1.328	0.000	1.213	1.143-1.289	0.000

OR odds ratio, *CI* confidence interval, *SIRS* systemic inflammatory response syndrome, *PT* prothrombin time, *SOFA* sequential organ failure assessment

Total SOFA is scored by 4 items (respiratory SOFA, cardiovascular SOFA, hepatic SOFA, renal SOFA).

For peer review only

Table 3. Scoring for the diagnosis of Sepsis-Induced Coagulopathy (SIC)

Category	Parameter	Score 0	Score 1	Score 2
Prothrombin time	PT-INR	≦ 1.2	> 1.2	> 1.4
Coagulation	Platelet count (x 10^3 /μL)	≧ 150	< 150	< 100
Total SOFA	SOFA 4 items	0	1	≧ 2

Diagnosed as Sepsis-Induced Coagulopathy (SIC) when the total score is 4 or more with total score of Prothrombin time and Coagulation exceed 2. Total SOFA is scored by 4 items (respiratory SOFA, cardiovascular SOFA, hepatic SOFA, renal SOFA). The score of each organ is defined as 2 in case of 2 or more.

PT prothrombin time, *INR* international normalization ratio, *SOFA* sequential organ failure assessment

For peer review only

For peer review only - http://bmjopen.bmj.com/site/about/guidelines.xhtml

Table 4. Patient count and mortality

		SIC		
		+	-	total
JAAM-DIC	+	855 (38.6%)	477 (27.7%)	1332 (34.7%)
	-	47 (34.0%)	119 (21.8%)	166 (25.3%)
	total	902 (38.4%)	596 (26.5%)	1498 (33.6%)

SIC sepsis-induced coagulopathy, *JAAM-DIC* Japanese Association for Acute Medicine-disseminated intravascular coagulation

For peer review only

Figure 1

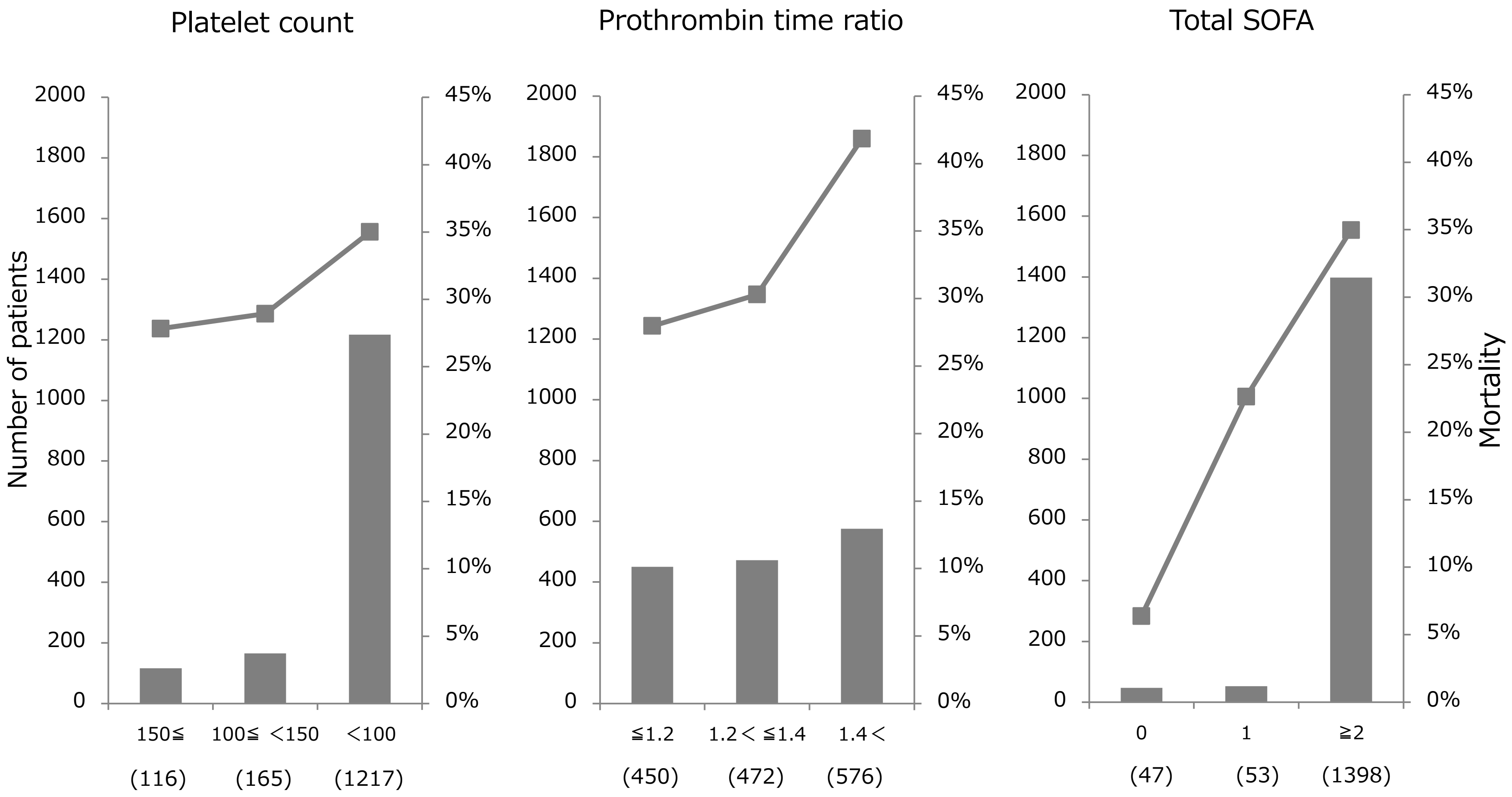
Platelet count
Prothrombin time ratio
Total SOFA
Number of patients
Mortality
2000
1800
1600
1400
1200
1000
800
600
400
200
0
45%
40%
35%
30%
25%
20%
15%
10%
5%
0%
150≦
100≦ ＜150
＜100
(116)
(165)
(1217)
≦1.2
1.2＜ ≦1.4
1.4＜
(450)
(472)
(576)
0
1
≧2
(47)
(53)
(1398)

22

Figure 2

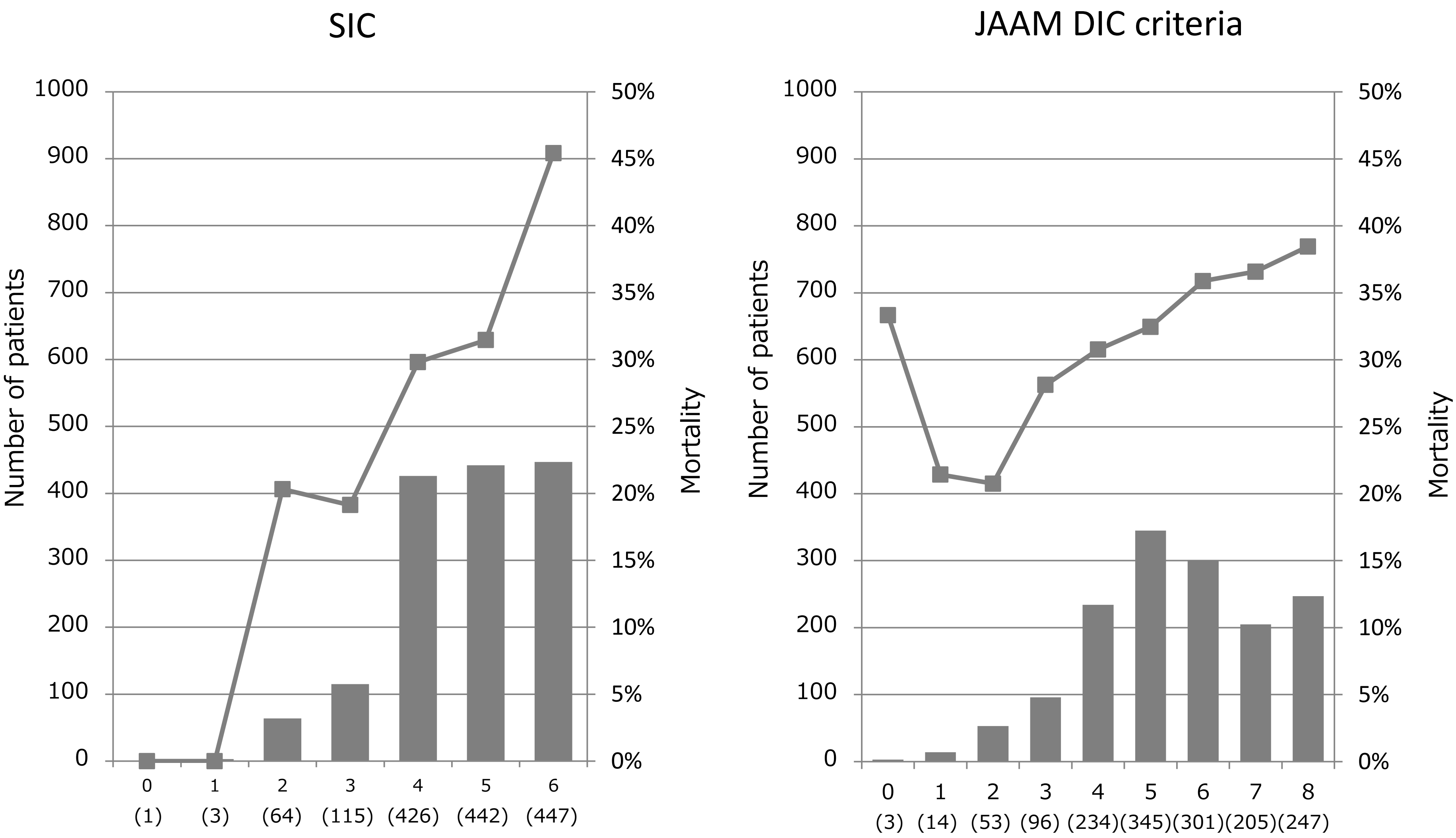
SIC
JAAM DIC criteria
Number of patients
Mortality
1000
900
800
700
600
500
400
300
200
100
0
50%
45%
40%
35%
30%
25%
20%
15%
10%
5%
0%
0 (1)
1 (3)
2 (64)
3 (115)
4 (426)
5 (442)
6 (447)
0 (3)
1 (14)
2 (53)
3 (96)
4 (234)
5 (345)
6 (301)
7 (205)
8 (247)

TRIPOD Checklist: Prediction Model Development

Section/Topic	Item	Checklist Item	Page
**Title and abstract**			
Title	1	Identify the study as developing and/or validating a multivariable prediction model, the target population, and the outcome to be predicted.	1
Abstract	2	Provide a summary of objectives, study design, setting, participants, sample size, predictors, outcome, statistical analysis, results, and conclusions.	3-4
**Introduction**			
Background and objectives	3a	Explain the medical context (including whether diagnostic or prognostic) and rationale for developing or validating the multivariable prediction model, including references to existing models.	5
	3b	Specify the objectives, including whether the study describes the development or validation of the model or both.	5
**Methods**			
Source of data	4a	Describe the study design or source of data (e.g., randomized trial, cohort, or registry data), separately for the development and validation data sets, if applicable.	6
	4b	Specify the key study dates, including start of accrual; end of accrual; and, if applicable, end of follow-up.	6
Participants	5a	Specify key elements of the study setting (e.g., primary care, secondary care, general population) including number and location of centres.	6
	5b	Describe eligibility criteria for participants.	6
	5c	Give details of treatments received, if relevant.	7
Outcome	6a	Clearly define the outcome that is predicted by the prediction model, including how and when assessed.	7
	6b	Report any actions to blind assessment of the outcome to be predicted.	7
Predictors	7a	Clearly define all predictors used in developing or validating the multivariable prediction model, including how and when they were measured.	7
	7b	Report any actions to blind assessment of predictors for the outcome and other predictors.	7
Sample size	8	Explain how the study size was arrived at.	NA
Missing data	9	Describe how missing data were handled (e.g., complete-case analysis, single imputation, multiple imputation) with details of any imputation method.	NA
Statistical analysis methods	10a	Describe how predictors were handled in the analyses.	7
	10b	Specify type of model, all model-building procedures (including any predictor selection), and method for internal validation.	7
	10d	Specify all measures used to assess model performance and, if relevant, to compare multiple models.	7
Risk groups	11	Provide details on how risk groups were created, if done.	NA
**Results**			
Participants	13a	Describe the flow of participants through the study, including the number of participants with and without the outcome and, if applicable, a summary of the follow-up time. A diagram may be helpful.	8
	13b	Describe the characteristics of the participants (basic demographics, clinical features, available predictors), including the number of participants with missing data for predictors and outcome.	8
Model development	14a	Specify the number of participants and outcome events in each analysis.	8-9
	14b	If done, report the unadjusted association between each candidate predictor and outcome.	NA
Model specification	15a	Present the full prediction model to allow predictions for individuals (i.e., all regression coefficients, and model intercept or baseline survival at a given time point).	10
	15b	Explain how to the use the prediction model.	10
Model performance	16	Report performance measures (with CIs) for the prediction model.	10
**Discussion**			
Limitations	18	Discuss any limitations of the study (such as nonrepresentative sample, few events per predictor, missing data).	13
Interpretation	19b	Give an overall interpretation of the results, considering objectives, limitations, and results from similar studies, and other relevant evidence.	13
Implications	20	Discuss the potential clinical use of the model and implications for future research.	13
**Other information**			
Supplementary information	21	Provide information about the availability of supplementary resources, such as study protocol, Web calculator, and data sets.	15
Funding	22	Give the source of funding and the role of the funders for the present study.	14

We recommend using the TRIPOD Checklist in conjunction with the TRIPOD Explanation and Elaboration document.

For peer review only

BMJ Open

New Criteria for Sepsis-induced coagulopathy (SIC) following the revised sepsis definition: a retrospective analysis of a nationwide survey.

Journal:	*BMJ Open*
Manuscript ID	bmjopen-2017-017046.R1
Article Type:	Research
Date Submitted by the Author:	05-Jul-2017
Complete List of Authors:	Iba, Toshiaki; Juntendo University Graduate School of Medicine, Department of Emergency and Disaster Med<br>Di Nisio, Marcello; University "G. D'Annunzio" of Chieti-Pescara, Department of Medical, Oral and Biotechnological Sciences; Academic Medical Center, Department of Vascular Medicine<br>Levy, Jerrold; Duke University, Anesthesiology<br>Kitamura, Naoya; Asahi Kasei Pharma Corporation, Pharmaceuticals Sales Division<br>Thachil, Jecko; Manchester Royal Infirmary
**Primary Subject Heading**:	Emergency medicine
Secondary Subject Heading:	Diagnostics
Keywords:	disseminated intravascular coagulation, prothrombin time, platelet count, sepsis, thrombomodulin

SCHOLARONE™ Manuscripts

For peer review only

Revised on 2017/7/03

Research article: **bmjopen-2017-017046.R1**

New Criteria for Sepsis-induced coagulopathy (SIC) following the revised sepsis definition: a retrospective analysis of a nationwide survey

Toshiaki Iba, M.D.[1]; Marcello Di Nisio, M.D.[2]; Jerrold H. Levy, M.D.[3]; Naoya Kitamura, B.S.[4]; and Jecko Thachil, M.D.[5]

[1] Professor of Emergency and Disaster Medicine, Juntendo University Graduate School of Medicine, Tokyo, Japan, toshiiba@cf6.so-net.ne.jp

[2] Professor of Medicine and Ageing Sciences, University G D'Annunzio of Chieti-Pescara, Chieti, Italy, mdinisio@unich.it

[3] Professor of Anesthesiology and Surgery, Duke University School of Medicine, Durham, NC, jerrold.levy@duke.edu

[4] Chief of Recomodulin Strategy Planning Department, Pharmaceuticals Sales Division, Asahi Kasei Pharma Corporation, Tokyo, Japan, kitamura.nb@om.asahi-kasei.co.jp

[5] Consultant Haematologist, Manchester Royal Infirmary, Manchester, UK, Jecko.thachil@cmft.nhs.uk

For peer review only

Corresponding author: Toshiaki Iba, MD.

Professor of Emergency and Disaster Medicine, Juntendo University Graduate School of Medicine

2-1-1 Hongo Bunkyo-ku, Tokyo 113-8421, Japan

E-mail: toshiiba@cf6.so-net.ne.jp, Tel: 81-3-3813-3111 (X: 5818) Fax: 81-3-3813-5431

Strength of this study

- Sepsis-induced coagulopathy (SIC) is the first scoring system specifically designed for coagulation disturbances in sepsis following the new Sepsis-3 definition.
- SIC is defined by the routine coagulation tests such as platelet count and PT ratio together with SOFA score.

Limitations

- Selection bias can exist since all the subjects were treated with recombinant thrombomodulin.
- Evaluation of the prognostic value of the SIC sore in patients receiving no anticoagulant has not yet been performed.

Short title: Proposal of new criteria for sepsis and coagulopathy

For peer review only - http://bmjopen.bmj.com/site/about/guidelines.xhtml

Abstract

OBJECTIVE: Recent clinical studies have shown that anticoagulant therapy might be effective only in specific at-risk subgroups of patients with sepsis and coagulation dysfunction. The definition of sepsis was recently modified and as such, old scoring systems may no longer be appropriate or the diagnosis of sepsis-associated coagulopathy. The aim of this study was to evaluate prognostic factors in patients diagnosed with sepsis and coagulopathy according to the new sepsis definition and assess their accuracy in comparison with existing models.

DESIGN: Retrospective analysis of the nationwide survey for recombinant human soluble thrombomodulin.

SETTING: General emergency and critical care centers in secondary and tertiary care hospitals.

PARTICIPANTS: We evaluated the prognostic value of the newly proposed diagnostic criteria for sepsis-induced coagulopathy. A total of 1498 Japanese patients with sepsis and coagulopathy complications who were treated with recombinant thrombomodulin were analyzed in this study.

MAIN OUTCOME MEASURES: The platelet count, prothrombin time (PT) ratio, fibrinogen and fibrin degradation products (FDP), systemic inflammatory response syndrome (SIRS) score, and sequential organ failure assessment (SOFA) score obtained just before the start of treatment were examined in relation to the 28-day mortality rate.

For peer review only - http://bmjopen.bmj.com/site/about/guidelines.xhtml

RESULTS: The platelet count, PT ratio, and total SOFA were independent predictors of a fatal outcome in a logistic regression model. A sepsis-induced coagulation (SIC) score was defined using the 3 above-mentioned variables with a positivity threshold of 4 points or more. The SIC score predicted higher 28-day mortality rate compared to the current Japanese Association for Acute Medicine (JAAM)-DIC score (38.4% vs. 34.7%).

CONCLUSIONS: The SIC score is based on readily available parameters, is easy to calculate and has a high predictive value for 28-day mortality. Future studies are warranted to evaluate whether the SIC score may guide the decision to initiate anticoagulant therapy.

Keywords

Disseminated intravascular coagulation; prothrombin time; platelet count; sepsis; thrombomodulin

For peer review only

For peer review only - http://bmjopen.bmj.com/site/about/guidelines.xhtml

Introduction

Shock and disseminated intravascular coagulation (DIC) are the two major causes of organ dysfunction in sepsis [1]. Dhainaut et al. reported that DIC is a strong predictor of mortality in septic patients independent of the severity of sepsis [2]. Although advances have been made in recent years in the management of septic shock leading to significant improvements in survival, less attention has been paid to the DIC component [3,4].

Although diagnostic criteria exist for DIC, none were specially designed for sepsis-associated DIC [5-7] that is uniquely characterized by coagulation activation with over-suppression of fibrinolysis and a high incidence of organ dysfunction [8]. The objectives of diagnostic criteria should ideally be to identify a homogenous group of patients with similar pathophysiology and clinical characteristics, whose outcomes can be improved by providing specific treatment interventions [9]. To reach these goals, diagnostic criteria should meet the following three conditions: (1) they should be readily available and easy to use; (2) they should enable diagnostic accuracy; and (3) they should have prognostic value. Since the new definition for sepsis was announced in 2016 [10], a DIC score that matches this definition and would help in identifying patients who would benefit from anticoagulant treatment is urgently needed. Therefore, the aim of this study was to evaluate prognostic factors in patients diagnosed with sepsis and coagulopathy according to the new sepsis definition and assess their accuracy in comparison with the existing models.

For peer review only

For peer review only - http://bmjopen.bmj.com/site/about/guidelines.xhtml

Methods

Data collection

The data set was obtained from a post-marketing survey examining recombinant human soluble thrombomodulin (TM-α; Asahi Kasei Parma Corporation, Tokyo, Japan) performed by Asahi Kasei Pharma Corporation between May 2008 and March 2010 [11] and kindly provided by the Japanese Society on Thrombosis and Hemostasis with permission. All of the cases treated in Japan during this period were registered in the survey. The survey was performed under the supervision of the Japanese Ministry of Health, Labour and Welfare (JMHW), and was conducted in accordance with the Declaration of Helsinki and Good Vigilance Practice and Good Post-marketing Study Practice. Since complete anonymization of personal data was performed upon data collection and the identification of the each patient was not possible, the ethical committees waived the need to acquire informed consent from patients.

At the time of data collection, sepsis was defined according to the American College of Chest Physicians/Society of Critical Care Medicine consensus definition [12]. A total of 2516 Japanese patients with sepsis-associated coagulation disorder were registered in this survey; however, since the record of sequential organ failure assessment (SOFA) data was not mandatory, a complete data set was only obtained in 1498 cases, and all of these patients were analyzed in this study. All patients were treated with TM-α (0.06 mg/kg/day for 6 days) by either intravenous bolus injection or intravenous infusion (diluted in 50 mL

For peer review only - http://bmjopen.bmj.com/site/about/guidelines.xhtml

0.9% saline) over 15 minutes via a catheter; the exclusion criteria were as follows: age less than 18 years, major bleeding, systemic inflammatory response syndrome (SIRS) score ≦ 1, hypersensitivity to TM-α, and pregnancy.

Laboratory measurements and organ dysfunction assessments

Blood samples obtained just before the initiation of anticoagulant therapy were analyzed and this data were defined as the 'initial data'. The platelet count, fibrinogen/fibrin degradation products (FDP), and prothrombin time (PT)-international normalization ratio (INR) were measured in local laboratories. The Japanese Association for Acute Medicine (JAAM)-DIC score [7] was calculated based on the initial (just before TM-α treatment) laboratory data and the SIRS score. Dysfunctions of the respiratory, cardiovascular, hepatic, and renal systems were assessed as in the SOFA score [13]. A score of 2 or more within each of these systems was defined as organ dysfunction.

Statistical analysis

Differences in patient characteristics between survivors and non-survivors were examined using the Fisher exact test or unpaired Wilcoxon signed-rank test. Then, the relationships between the 28-day mortality rate and the initial data were examined in univariate and multivariate analyses using logistic regression analysis (the enter method). The analysis was conducted using the outcome (survived, 0; died, 1) as the dependent variable and age, sex, SIRS score, platelet count, PT ratio, and the proportion of various organ dysfunction as explanatory variables.

For peer review only - http://bmjopen.bmj.com/site/about/guidelines.xhtml

The numerical values in the text and tables represent the median and interquartile range (IQR), unless otherwise noted. The results of the logistic regression analysis were reported as the odds ratio (OR), 95% confidence interval (CI) and *P*-values. For all the reported results, a value of $P < 0.05$ was considered to denote statistical significance. The above-mentioned analyses were performed using JMP software, version 9.0 (SAS Institute Co., Ltd., Cary, North Carolina).

Results

Baseline characteristics

Among the 1498 patients, 994 (66.4%) survived at 28 days and 504 (33.6%) died. Table 1 shows the baseline characteristics of the patients. The median age was lower and the proportion of women was larger among the survivors. No difference in the SIRS score (P = 0.0556) was seen between the two groups, but the JAAM-DIC score (P = 0.0019) was higher among the non-survivors. Regarding the hemostatic parameters, the platelet count was higher ($P < 0.0001$) and the PT ratio was lower ($P < 0.0001$) among the survivors. In contrast, the FDP level was not significantly different between the survivors and non-survivors. The initial total SOFA score was significantly higher among the non-survivors ($P < 0.0001$), and the proportions of patients with respiratory dysfunction ($P < 0.0001$), cardiovascular dysfunction (P = 0.0426), hepatic dysfunction ($P < 0.0001$), or renal dysfunction ($P < 0.0001$) were higher among the non-survivors.

Relationships between biomarkers and mortality

For peer review only - http://bmjopen.bmj.com/site/about/guidelines.xhtml

The results of the univariate and multivariable analyses are shown in Table 2. A multivariate analysis using the enter method showed that patient age (P = 0.002), male sex (P = 0.017), decreased platelet count (P = 0.005), higher PT ratio (P = 0.024), and higher total SOFA (P < 0.001) were significantly and independently associated with the 28-day mortality rate. In contrast, the initial SIRS score was not correlated with survival (Table 2).

The SIC score

The SIC score was developed based on the result of the logistic regression analyses. The relationship between the initial platelet count and mortality is shown in Figure 1, left panel. As shown in the figure, the mortality rate was less than 30% when the platelet count was more than 100 x 10^3 μL but increased to 35% when the platelet count decreased below 100 x 10^3 μL. In contrast, the mortality rate increased steadily as the initial PT ratio increased and reached 40% at a PT ratio of more than 1.4 (Figure 1, middle). The mortality increased along with the increase of the initial total SOFA (Figure 1, right). For the SIC scoring, the organ dysfunction scores were defined according to the criteria used for the SOFA score [13]. Total SOFA is calculated as the sum of the 4 items (respiratory SOFA, cardiovascular SOFA, hepatic SOFA, renal SOFA). The score of total SOFA was defined as 2 if the total score exceeded 2. For the PT ratio, the cutoff value to assign 1 point was set at 1.2 in accordance with the JAAM-DIC criteria, while the cutoff value for 2 points was set at 1.4 based on the case number distribution and the mortality rate. Finally, SIC was defined as a total score of 4 or more (Table 3), since the mortality rate at this point exceeded 20%

For peer review only - http://bmjopen.bmj.com/site/about/guidelines.xhtml

For peer review only

(Figure 2, left), with the requirement that the total score for the platelet count and the PT ratio exceed 2.

Comparison of the SIC and JAAM-DIC criteria

A total of 902 patients were diagnosed as having SIC, while 1332 patients met the JAAM criteria for DIC. The respective mortality rates for these classifications were 38.4% and 34.7%. Among the patients diagnosed with DIC by the JAAM score, 477 cases were negative with SIC while 47 patients were DIC negative using the JAAM-DIC but positive with SIC (Table 4). The mortality of the patients having positive JAAM-DIC but negative SIC was 27.7%; while that of patients positive with SIC but negative with JAAM-DIC was 34.0%. Figure 2 shows the relationship between the 28-day mortality rate and the SIC score (left panel) and the JAAM-DIC score (right panel). Using the SIC scoring system, the mortality rate increased in a linear fashion. The mortality rate was 30% at a score of 4 and increased steeply to a maximum of over 45% at a score of 6. In contrast, the mortality rate did not seem to be strongly correlated with the JAAM-DIC score. The mortality rate had already exceeded 20% at scores of 1 to 3, and it increased to over 30% at a score of 4. The mortality rate increased only gradually thereafter, reaching approximately 40% at a score of 8.

The median JAAM-DIC score in the survivors was 5 (4 to 7) before treatment and it decreased to 3 (1 to 4). The score also decreased from 6 (5 to 7) to 5 (4 to 6) in the

For peer review only - http://bmjopen.bmj.com/site/about/guidelines.xhtml

non-survivors. In contrast, though SIC score decreased from 5 (4 to 5) to 3 (2 to 3) in survivors, it did not decrease in non-survivors (5 [4 to 6] to 5 [3 to 5]).

Discussion

The relationship and balance between coagulation and fibrinolysis system is regulated by a complex series of interactions. Microbial products induce the synthesis and the release of inflammatory mediators called pathogen-associated molecular patterns (PAMPs) during sepsis. In addition to these PAMPs, pro-inflammatory substances known as damage-associated molecular patterns (DAMPs) are also released from activated or damaged host cells [14,15]. Both PAMPs and DAMPs activate the coagulation system together with the host immune response, leading to DIC [14,16]. Since the primary function of these biologic responses is to assist the host in sequestering and eliminating the microbes, the suppression of coagulation at this stage may not always lead to a better outcome. In contrast, patients with compromised organ circulation may benefit from anticoagulant therapy [17]. However, all of the anticoagulants examined in the early 21st century failed to show any efficacy in these patients [18,19]. One of the major reasons for this was that these studies targeted severe sepsis (without coagulation disturbances), rather than sepsis-associated coagulopathy [2,20]. After a series of disappointing reports, Umemura et al. suggested that anticoagulant therapies might be beneficial if they were applied to septic patients who have a severe coagulation disorder [21]. In addition, Yamakawa et al. also

For peer review only - http://bmjopen.bmj.com/site/about/guidelines.xhtml

reported an association between the application of anticoagulant therapy and a decrease in mortality among patients with a high risk of death (SOFA score of 13–17) [22]. With respect to recombinant thrombomodulin, recent studies examining its effects in patients with septic DIC have repeatedly reported favorable results [2,22] with more prominent benefits as the severity of coagulopathy increased [23,24]. In a Phase 2 clinical trial performed in 17 countries, recombinant thrombomodulin tended to exhibit an effect in patients with organ dysfunction having a PT ratio of greater than 1.4 before the treatment [24]. Recombinant thrombomodulin has been used in Japanese patients who meet the JAAM-DIC criteria which, however, might not be appropriate [25]. Yoshimura et al. [26] reported that a beneficial effect was only seen in patients diagnosed as having DIC and had an APACHE II score of between 24 and 29. Thus, to identify appropriate candidates for anticoagulant therapy, we added a category for organ dysfunction in patients with SIC and targeted individuals with a mortality rate higher than that of patients meeting the JAAM-DIC criteria. This new SIC category represents "sepsis (Third International Consensus Definitions [Sepsis 3] [10]), and coagulation disorder," which could be a suitable target for therapeutic interventions. Previous reports have consistently reported efficacy when the 30-day mortality rate in the TM-α-treated group was between 20% and 30% [27,28], and not below 20% [24]. As a matter of fact, a subgroup analysis of a Phase 3 study performed in Japan demonstrated that the mortality rate in a heparin-control group was 31.6%, while that of TM-α group was 21.4% [29]. In the present study, the mortality

For peer review only - http://bmjopen.bmj.com/site/about/guidelines.xhtml

rate of the SIC group was about 30% when the total score was 4, and it increased as the SIC score increased.

Another purpose of this study was to compare the SIC and JAAM-DIC criteria. In SIC, the SIRS score used in the JAAM-DIC criteria was replaced with the SOFA score, and the FDP criterion was eliminated. This was based on the fact that the prognostic relevance of SIRS has been questioned and was not used in the new definition of sepsis. Besides, the significance of fibrin-related markers in DIC differs depending on the underlying disease [30], and the impact of the FDP criterion was limited to patients with fibrinolysis-suppressed-type coagulation disorders, as represented by sepsis. The current study indicated that the SIC score identified patients with a higher risk of death, compared with the JAAM-DIC score (38.4% vs. 34.7%), and the numbers of diagnosed cases were 902 and 1332, respectively. The mortality of patients who satisfied SIC criteria but not JAAM-DIC criteria was 6.3% higher than that diagnosed using JAAM-DIC criteria but not satisfied SIC criteria. Therefore, we speculate that the SIC score might better identify candidates for anticoagulant therapy rather than the JAAM-DIC score. The other advantage of SIC is the simplicity of its calculation. Indeed, the FDP criterion was omitted, and SIRS score was replaced by the SOFA score. Since the SOFA score is routinely evaluated in the ICU, the addition of a PT test is relatively easy to implement in clinical practice.

Limitations

For peer review only - http://bmjopen.bmj.com/site/about/guidelines.xhtml

There are limitations to our current study. First, data from a post-marketing survey was utilized in this study and all subjects were treated with recombinant thrombomodulin. While treatment could influence the overall 28-day mortality rate, it is unlikely that it affected the performance of the SIC score. This study also did not analyze the ability of SIC in identifying septic patients with coagulopathy not treated with any anticoagulants, or treated with anticoagulants other than TM-α, such as antithrombin.

Conclusions

We have proposed SIC as a new score for patients with "sepsis and coagulopathy." Since the SIC score adheres to the new sepsis criteria (Third International Consensus Definitions), this definition will be easy to use and should provide important and novel information to the physicians.

Acknowledgement

The authors would like to thank all the institutes that participated in the post-marketing surveillance.

Contributors

For peer review only

TI, MDN, and JT conceived of the study, and participated in its design. TI and MDN participated in the sequence alignment and drafted the manuscript. JL helped to revise the manuscript. NK helped to collect and arrange the data. All authors read and approved the final manuscript.

Funding

This work was supported by Ministry of Education, Culture, Sports, Science and Technology-Supported Program for the Strategic Research Foundation at Private Universities 2016.

Competing interests

KN is an employee of Asahi Kasei Pharma Corporation. The other authors have no competing interests to declare.

Data sharing statement

Full data and data analysis files are available on request.

Supplementary resources

None

For peer review only

References

1. Gando S, Levi M, Toh CH: **Disseminated intravascular coagulation.** *Nat Rev Dis Primers* 2016, **2**:16037.

2. Dhainaut JF, Yan SB, Joyce DE, Pettilä V, Basson B, Brandt JT, Sundin DP, Levi M: **Treatment effects of drotrecogin alfa (activated) in patients with severe sepsis with or without overt disseminated intravascular coagulation.** *J Thromb Haemost* 2004, **2**:1924-1933.

3. Kadri SS, Rhee C, Strich JR, Morales MK, Hohmann S, Menchaca J, Suffredini AF, Danner RL, Klompas M: **Estimating Ten-Year Trends in Septic Shock Incidence and Mortality in United States Academic Medical Centers Using Clinical Data.** *Chest* 2016, **151**(2):278-285.

4. Dellinger RP, Levy MM, Rhodes A, Annane D, Gerlach H, Opal SM, Sevransky JE, Sprung CL, Douglas IS, Jaeschke R, *et al*: **Surviving sepsis campaign: international guidelines for management of severe sepsis and septic shock: 2012.** *Crit Care Med* 2013; **41**:580-637.

5. Kobayashi N, Maekawa T, Takada M, Tanaka H, Gonmori H: **Criteria for diagnosis of DIC based on the analysis of clinical and laboratory findings in 345 DIC patients collected by the Research Committee on DIC in Japan.** *Bibl Haematol* 1983, **49**:265-275.

For peer review only - http://bmjopen.bmj.com/site/about/guidelines.xhtml

6. Taylor FB Jr, Toh CH, Hoots WK, Wada H, Levi M: **Towards definition, clinical and laboratory criteria, and a scoring system for disseminated intravascular coagulation.** *Thromb Haemost* 2001, **86**:1327-1330.

7. Gando S, Iba T, Eguchi Y, Ohtomo Y, Okamoto K, Koseki K, Mayumi T, Murata A, Ikeda T, Ishikura H, et al: **A multicenter, prospective validation of disseminated intravascular coagulation diagnostic criteria for critically ill patients: comparing current criteria.** *Crit Care Med* 2006, **34**:625-631.

8. Levi M, Ten Cate H: **Disseminated intravascular coagulation.** *N Engl J Med* 1999, **341**:586-592.

9. Gando S, Meziani F, Levi M: **What's new in the diagnostic criteria of disseminated intravascular coagulation?** *Intensive Care Med* 2016; **42**:1062-1064.

10. Singer M, Deutschman CS, Seymour CW, Shankar-Hari M, Annane D, Bauer M, Bellomo R, Bernard GR, Chiche JD, Coopersmith CM, *et al*: **The Third International Consensus Definitions for Sepsis and Septic Shock (Sepsis-3).** *JAMA* 2016, **315**:801-810.

11. Mimuro J, Takahashi H, Kitajima I, Tsuji H, Eguchi Y, Matsushita T, Kuroda T, Sakata Y: **Impact of recombinant soluble thrombomodulin (thrombomodulin alfa) on disseminated intravascular coagulation.** *Thromb Res* 2013, **131**:436-443.

12. Bone RC, Balk RA, Cerra FB, Dellinger RP, Fein AM, Knaus WA, Schein RM, Sibbald WJ: **Definitions for sepsis and organ failure and guidelines for the use of**

For peer review only

For peer review only - http://bmjopen.bmj.com/site/about/guidelines.xhtml

innovative therapies in sepsis. The ACCP/SCCM Consensus Conference Committee. American College of Chest Physicians/Society of Critical Care Medicine. *Chest* 1992, **101**:1644-1655.

13. Vincent JL, de Mendon.a A, Cantraine F, Moreno R, Takala J, Suter PM, Sprung CL, Colardyn F, Blecher S: **Use of the SOFA score to assess the incidence of organ dysfunction/failure in intensive care units: results of a multicenter, prospective study.** *Crit Care Med* 1998, **26**:1793-1800.
14. Esmon CT, Xu J, Lupu F: **Innate immunity and coagulation.** *J Thromb Haemost* 2011, **9**:182-188.
15. Seong SY, Matzinger P: **Hydrophobicity: an ancient damage-associated molecular pattern that initiates innate immune responses.** *Nat Rev Immunol* 2004, **4**: 469**-4**78.
16. Liaw PC, Ito T, Iba T, Thachil J, Zeerleder S: **DAMP and DIC: The role of extracellular DNA and DNA-binding proteins in the pathogenesis of DIC.** *Blood Rev* 2016, **30**:257-261.
17. Schouten M, Wiersinga WJ, Levi M, van der Poll T: **Inflammation, endothelium, and coagulation in sepsis.** *J Leukoc Biol* 2008, **83**:536-545.
18. Warren BL, Eid A, Singer P, Pillay SS, Carl P, Novak I, Chalupa P, Atherstone A, Pénzes I, Kübler A, et al: **Caring for the critically ill patient. High-dose antithrombin III in severe sepsis: a randomized controlled trial.** *JAMA* 2001, **286**:1869-1878.

For peer review only - http://bmjopen.bmj.com/site/about/guidelines.xhtml

19. Ranieri VM, Thompson BT, Barie PS, Dhainaut JF, Douglas IS, Finfer S, Gårdlund B, Marshall JC, Rhodes A, Artigas A, et al: **Drotrecogin alfa (activated) in adults with septic shock.** *N Engl J Med* 2012, **366**:2055-2064.

20. Kienast J, Juers M, Wiedermann CJ, Hoffmann JN, Ostermann H, Strauss R, Keinecke HO, Warren BL, Opal SM: **Treatment effects of high-dose antithrombin without concomitant heparin in patients with severe sepsis with or without disseminated intravascular coagulation.** *J Thromb Haemost* 2006, **4**:90-97.

21. Umemura Y, Yamakawa K, Ogura H, Yuhara H, Fujimi S: **Efficacy and safety of anticoagulant therapy in three specific populations with sepsis: a meta-analysis of randomized controlled trials.** *J Thromb Haemost* 2015, **14**:518-530.

22. Yamakawa K, Umemura Y, Hayakawa M, Kudo D, Sanui M, Takahashi H, Yoshikawa Y, Hamasaki T, Fujimi S: **Benefit profile of anticoagulant therapy in sepsis: a nationwide multicentre registry in Japan.** *Crit Care* 2016, **20**:229.

23. Yamakawa K, Aihara M, Ogura H, Yuhara H, Hamasaki T, Shimazu T: **Recombinant human soluble thrombomodulin in severe sepsis: a systematic review and meta-analysis.** *J Thromb Haemost* 2015, **13**:508-519.

24. Vincent JL, Ramesh MK, Ernest D, LaRosa SP, Pachl J, Aikawa N: **A randomized, double-blind, placebo-controlled, Phase 2b study to evaluate the safety and efficacy of recombinant human soluble thrombomodulin, ART-123, in patients with sepsis and suspected disseminated intravascular coagulation.** *Crit Care Med* 2013, **41**:2069-2079.

For peer review only - http://bmjopen.bmj.com/site/about/guidelines.xhtml

25. Tagami T, Matsui H, Horiguchi H, Fushimi K, Yasunaga H: **Recombinant human soluble thrombomodulin and mortality in severe pneumonia patients with sepsis-associated disseminated intravascular coagulation: an observational nationwide study.** *J Thromb Haemost* 2015, **13**:31-40.

26. Yoshimura J, Yamakawa K, Ogura H, Umemura Y, Takahashi H, Morikawa M, Inoue Y, Fujimi S, Tanaka H, Hamasaki T, et al: **Benefit profile of recombinant human soluble thrombomodulin in sepsis-induced disseminated intravascular coagulation: a multicenter propensity score analysis.** *Crit Care* 2015, **19**:78.

27. Yamakawa K, Ogura H, Fujimi S, Morikawa M, Ogawa Y, Mohri T, Nakamori Y, Inoue Y, Kuwagata Y, Tanaka H, et al: **Recombinant human soluble thrombomodulin in sepsis-induced disseminated intravascular coagulation: a multicenter propensity score analysis.** *Intensive Care Med* 2013, **39**:644-652.

28. Hayakawa M, Yamakawa K, Saito S, Uchino S, Kudo D, Iizuka Y, Sanui M, Takimoto K, Mayumi T, Ono K: **Recombinant human soluble thrombomodulin and mortality in sepsis-induced disseminated intravascular coagulation. A multicentre retrospective study.** *Thromb Haemost* 2016, **115**:1157-66.

29. Aikawa N, Shimazaki S, Yamamoto Y, Saito H, Maruyama I, Ohno R, Hirayama A, Aoki Y, Aoki N: **Thrombomodulin alfa in the treatment of infectious patients complicated by disseminated intravascular coagulation: subanalysis from the phase 3 trial.** *Shock* 2011, **35**:349-354.

For peer review only - http://bmjopen.bmj.com/site/about/guidelines.xhtml

30. Wada H, Matsumoto T, Yamashita Y, Hatada T: **Disseminated intravascular coagulation: testing and diagnosis.** *Clin Chim Acta* 2014, **436**:130-134.

For peer review only

Legends

Figure 1. Patient counts and mortality rates according to platelet count, prothrombin time ratio, and total SOFA score. The bar graph shows the number of patients in each category, and the line graph represents the mortality rate. X-axis represents the score and (case number). Left: Many of the patients had an initial platelet count of 100 x 10^3 µL or less. Mortality increased to 35% when the count decreased to less than 100 x 10^3 µL. Middle: Mortality increased along with an increase in the prothrombin time ratio, reaching more than 40% when the prothrombin time ratio was more than 1.4. Right: The population of total SOFA score of 0 and 1 are quite limited, and the mortality of this population was lower than that of the score of 2 or more.

Figure 2. Patient counts and mortality rates according to the sepsis-induced coagulopathy (SIC) and the Japanese Association for Acute Medicine (JAAM) disseminated intravascular coagulation (DIC) classifications. The patient distributions (bars) and the mortality rates (lines) are plotted according to the SIC scores (left) and the JAAM-DIC scores (right). X-axis represents the score and (case number). The mortality rate increased as SIC score elevated and exceeded 20% at a score of 4. In contrast, the mortality rate exceeded 30% at a JAAM-DIC score of 4 and gradually increased to 40%.

For peer review only - http://bmjopen.bmj.com/site/about/guidelines.xhtml

Table 1. Baseline characteristics of the patients

Characteristics	Survivor (n = 994)	Non-survivor (n = 504)	*P*-value
Age (years)	70 (58–78)	73 (62–80)	0.0001
Sex (male/female)	560/434	329/175	0.0009
Baseline values			
SIRS score	3 (2–4)	3 (2–4)	0.0556
SIRS score ≧3	625(62.9%)	341(67.7%)	0.0677
JAAM-DIC score	5 (4–7)	6 (5–7)	0.0019
Platelet count (x 10^3 μL)	61 (36–89)	49 (29–78)	< 0.0001
FDP (μg/mL)	25.3 (13.0–51.9)	25.4 (12.2–51.7)	0.7788
PT ratio	1.30 (1.16-1.48)	1.36 (1.21–1.64)	< 0.0001
Organ dysfunction			
Total SOFA	5 (3–6)	5 (4–7)	< 0.0001
respiratory SOFA ≧2* (PaO_2/FiO_2 < 300)	621 (62.5%)	395 (78.4%)	< 0.0001
cardiovascular SOFA ≧2* (requirement of vasopressors)	636 (64.0%)	349 (69.3%)	0.0426
hepatic SOFA ≧2* (bilirubin ≧2.0 mg/dL)*	272 (27.4%)	202 (40.8%)	< 0.0001
renal SOFA ≧2* (creatinine ≧2.0 mg/dL)	326 (32.8%)	221 (43.8%)	< 0.0001

JAAM Japanese association for acute medicine, *DIC* disseminated intravascular coagulation, *SIRS* systemic inflammatory response syndrome, *FDP* fibrinogen and fibrin degradation products, *PT* prothrombin time, *SOFA* sequential organ failure assessment

Total SOFA is the sum the 4 items (respiratory SOFA, cardiovascular SOFA, hepatic SOFA, renal SOFA). The score of total SOFA is defined as 2 if the total score exceeded 2.

* Defined according to the Third International Consensus for Sepsis and Septic Shock. [13].

For peer review only

Table 2. Relationship between 28-day mortality and baseline characteristics

Item	Univariate			Multivariate		
	OR	95% CI	*P*-value	OR	95% CI	*P*-value
Age (years)	1.010	1.004-1.016	0.002	1.010	1.004-1.017	0.002
Sex (male/female)	1.457	1.168-1.821	0.001	1.323	1.052-1.668	0.017
Platelet count (x10^9/L)	0.965	0.945-0.984	0.000	0.972	0.951-0.992	0.005
PT ratio	1.225	1.065-1.435	0.004	1.169	1.020-1.364	0.024
Total SOFA	1.252	1.181-1.328	0.000	1.213	1.143-1.289	0.000

OR odds ratio, *CI* confidence interval, *SIRS* systemic inflammatory response syndrome, *PT* prothrombin time, *SOFA* sequential organ failure assessment

Total SOFA is the sum the 4 items (respiratory SOFA, cardiovascular SOFA, hepatic SOFA, renal SOFA). The score of total SOFA is defined as 2 if the total score exceeded 2.

For peer review only

Table 3. Scoring for the diagnosis of Sepsis-Induced Coagulopathy (SIC)

Category	Parameter	0 point	1 point	2 points
Prothrombin time	PT-INR	≦ 1.2	> 1.2	> 1.4
Coagulation	Platelet count (x 10^3 /μL)	≧ 150	< 150	< 100
Total SOFA	SOFA 4 items	0	1	≧ 2

Diagnosed as Sepsis-Induced Coagulopathy (SIC) when the total score is 4 or more with total score of Prothrombin time and Coagulation exceed 2.

Total SOFA is the sum the 4 items (respiratory SOFA, cardiovascular SOFA, hepatic SOFA, renal SOFA). The score of total SOFA is defined as 2 if the total score exceeded 2.

PT prothrombin time, *INR* international normalization ratio, *SOFA* sequential organ failure assessment

For peer review only - http://bmjopen.bmj.com/site/about/guidelines.xhtml

Table 4. Patient count and mortality

		SIC		
		+	-	total
JAAM-DIC	+	855 (38.6%)	477 (27.7%)	1332 (34.7%)
	-	47 (34.0%)	119 (21.8%)	166 (25.3%)
	total	902 (38.4%)	596 (26.5%)	1498 (33.6%)

SIC sepsis-induced coagulopathy, *JAAM-DIC* Japanese Association for Acute Medicine-disseminated intravascular coagulation

For peer review only

Figure 1

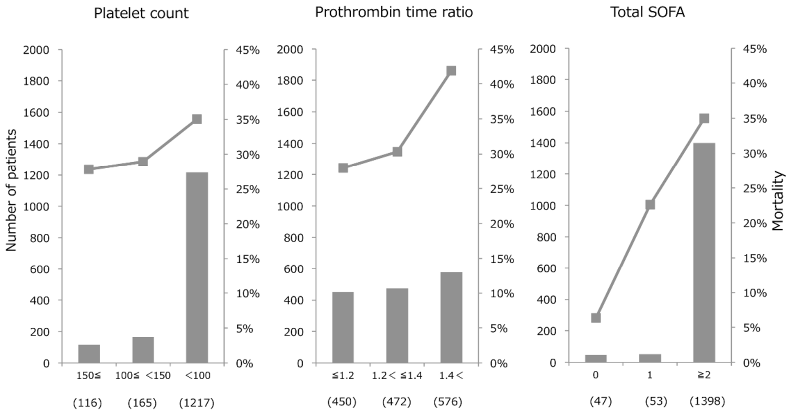

27

297x209mm (300 x 300 DPI)

view only

Figure 2

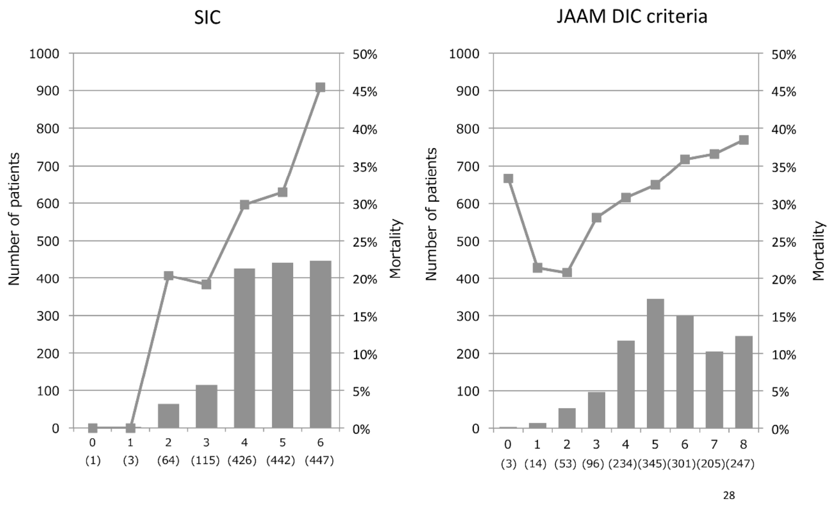

297x209mm (300 x 300 DPI)

view only

TRIPOD Checklist: Prediction Model Development

Section/Topic	Item	Checklist Item	Page
**Title and abstract**			
Title	1	Identify the study as developing and/or validating a multivariable prediction model, the target population, and the outcome to be predicted.	1
Abstract	2	Provide a summary of objectives, study design, setting, participants, sample size, predictors, outcome, statistical analysis, results, and conclusions.	3-4
**Introduction**			
Background and objectives	3a	Explain the medical context (including whether diagnostic or prognostic) and rationale for developing or validating the multivariable prediction model, including references to existing models.	5
	3b	Specify the objectives, including whether the study describes the development or validation of the model or both.	5
**Methods**			
Source of data	4a	Describe the study design or source of data (e.g., randomized trial, cohort, or registry data), separately for the development and validation data sets, if applicable.	6
	4b	Specify the key study dates, including start of accrual; end of accrual; and, if applicable, end of follow-up.	6
Participants	5a	Specify key elements of the study setting (e.g., primary care, secondary care, general population) including number and location of centres.	6
	5b	Describe eligibility criteria for participants.	6
	5c	Give details of treatments received, if relevant.	7
Outcome	6a	Clearly define the outcome that is predicted by the prediction model, including how and when assessed.	7
	6b	Report any actions to blind assessment of the outcome to be predicted.	7
Predictors	7a	Clearly define all predictors used in developing or validating the multivariable prediction model, including how and when they were measured.	7
	7b	Report any actions to blind assessment of predictors for the outcome and other predictors.	7
Sample size	8	Explain how the study size was arrived at.	NA
Missing data	9	Describe how missing data were handled (e.g., complete-case analysis, single imputation, multiple imputation) with details of any imputation method.	NA
Statistical analysis methods	10a	Describe how predictors were handled in the analyses.	7
	10b	Specify type of model, all model-building procedures (including any predictor selection), and method for internal validation.	7
	10d	Specify all measures used to assess model performance and, if relevant, to compare multiple models.	7
Risk groups	11	Provide details on how risk groups were created, if done.	NA
**Results**			
Participants	13a	Describe the flow of participants through the study, including the number of participants with and without the outcome and, if applicable, a summary of the follow-up time. A diagram may be helpful.	8
	13b	Describe the characteristics of the participants (basic demographics, clinical features, available predictors), including the number of participants with missing data for predictors and outcome.	8
Model development	14a	Specify the number of participants and outcome events in each analysis.	8-9
	14b	If done, report the unadjusted association between each candidate predictor and outcome.	NA
Model specification	15a	Present the full prediction model to allow predictions for individuals (i.e., all regression coefficients, and model intercept or baseline survival at a given time point).	10
	15b	Explain how to the use the prediction model.	10
Model performance	16	Report performance measures (with CIs) for the prediction model.	10
**Discussion**			
Limitations	18	Discuss any limitations of the study (such as nonrepresentative sample, few events per predictor, missing data).	13
Interpretation	19b	Give an overall interpretation of the results, considering objectives, limitations, and results from similar studies, and other relevant evidence.	13
Implications	20	Discuss the potential clinical use of the model and implications for future research.	13
**Other information**			
Supplementary information	21	Provide information about the availability of supplementary resources, such as study protocol, Web calculator, and data sets.	15
Funding	22	Give the source of funding and the role of the funders for the present study.	14

We recommend using the TRIPOD Checklist in conjunction with the TRIPOD Explanation and Elaboration document.

For peer review only
